# Reactive Oxygen Species Scavenging by Catalase Is Important for Female *Lutzomyia longipalpis* Fecundity and Mortality

**DOI:** 10.1371/journal.pone.0017486

**Published:** 2011-03-09

**Authors:** Hector Diaz-Albiter, Roanna Mitford, Fernando A. Genta, Mauricio R. V. Sant'Anna, Rod J. Dillon

**Affiliations:** 1 Vector Group, Liverpool School of Tropical Medicine, Liverpool, United Kingdom; 2 Instituto Oswaldo Cruz, Fundação Oswaldo Cruz and Instituto Nacional de Ciência e Tecnologia – Entomologia Molecular, Rio, Brazil; Instituto Butantan, Brazil

## Abstract

The phlebotomine sand fly *Lutzomyia longipalpis* is the most important vector of American visceral leishmaniasis (AVL), the disseminated and most serious form of the disease in Central and South America. In the natural environment, most female *L. longipalpis* are thought to survive for less than 10 days and will feed on blood only once or twice during their lifetime. Successful transmission of parasites occurs when a *Leishmania*-infected female sand fly feeds on a new host. Knowledge of factors affecting sand fly longevity that lead to a reduction in lifespan could result in a decrease in parasite transmission. Catalase has been found to play a major role in survival and fecundity in many insect species. It is a strong antioxidant enzyme that breaks down toxic reactive oxygen species (ROS). Ovarian catalase was found to accumulate in the developing sand fly oocyte from 12 to 48 hours after blood feeding. Catalase expression in ovaries as well as oocyte numbers was found to decrease with age. This reduction was not found in flies when fed on the antioxidant ascorbic acid in the sugar meal, a condition that increased mortality and activation of the prophenoloxidase cascade. RNA interference was used to silence catalase gene expression in female *Lu. longipalpis*. Depletion of catalase led to a significant increase of mortality and a reduction in the number of developing oocytes produced after blood feeding. These results demonstrate the central role that catalase and ROS play in the longevity and fecundity of phlebotomine sand flies.

## Introduction

The phlebotomine sand fly *Lutzomyia longipalpis*, Lutz and Neiva 1912 is the best studied and most important vector of American Visceral Leishmaniasis (AVL) [1], [2]. It is widely found in Latin America, from the South of Mexico to the North of Argentina [3], [4]. *Lu. longipalpis* is a permissive vector to *Leishmania* infections [5] and this together with its wide distribution in urban environments [6] makes this sand fly species an ideal model to study phlebotomine physiology to help develop future vector control methods for the disruption of *Leishmania* transmission.

Previous studies in other blood-feeding insect vectors have shown that reactive oxygen species (ROS) play an important role in both reproductive output [7] and survival [8], [9], [10], [11], [12]. Biological damage related to ROS production has also been implicated in the process of ageing in dipterans like *Drosophila melanogaster*, previous work done on this species showed that oxidative stress increases with age, while antioxidant enzyme activity decreased over time [13], [14], [15].

ROS are regularly generated by mitochondrial electron transport [16]. Partially reduced and highly reactive metabolites of O_2_ such as superoxide anion (O_2_^−^·) and hydrogen peroxide (H_2_O_2_) are formed during cellular respiration. These partially reduced metabolites of O_2_ are often referred to as reactive oxygen species due to their higher reactivity in relation to molecular oxygen [17]. Excessive release of ROS damages lipids, proteins, and DNA [18] which leads to oxidative stress, loss of cell function, and programmed cell death [19]. ROS are also actively released as a response against bacterial and parasitic pathogens in different insect species [8], [20], [21]. To regulate oxidative stress, the eukaryotic cell produces different ROS-scavenging enzymes, such as superoxide dismutase (which reduces O_2_^−^· to H_2_O_2_), glutathione peroxidase and catalase (which reduces H_2_O_2_ to H_2_O) [17]. Although many studies have been published regarding mechanisms to resist oxidative stress in *Leishmania* parasites [22], [23], [24], [25] there is little information on ROS-scavenging molecular mechanisms on sand flies, despite the fact that putative antioxidant enzymes have been found to be upregulated in two different species of phlebotomine sand flies upon *Leishmania* infection [26], [27].

In order to investigate the biological role of ROS-scavenging in fecundity and survival, we analysed the expression of catalase in three different age groups of female sand flies. Fecundity and catalase expression decreased with age. Catalase was incriminated as an important component in the loss of fecundity using RNAi. Dietary supplementation with an exogenous ROS-scavenger was found to partially reverse the differences in fecundity and increase mortality with a concomitant activation of the phenoloxidase (PO) cascade. These results show that both fecundity and survival are affected by endogenous and exogenous ROS-scavenging in female *Lutzomyia longipalpis*.

## Results

### Age-related decrease of fecundity

**Figure 1:**
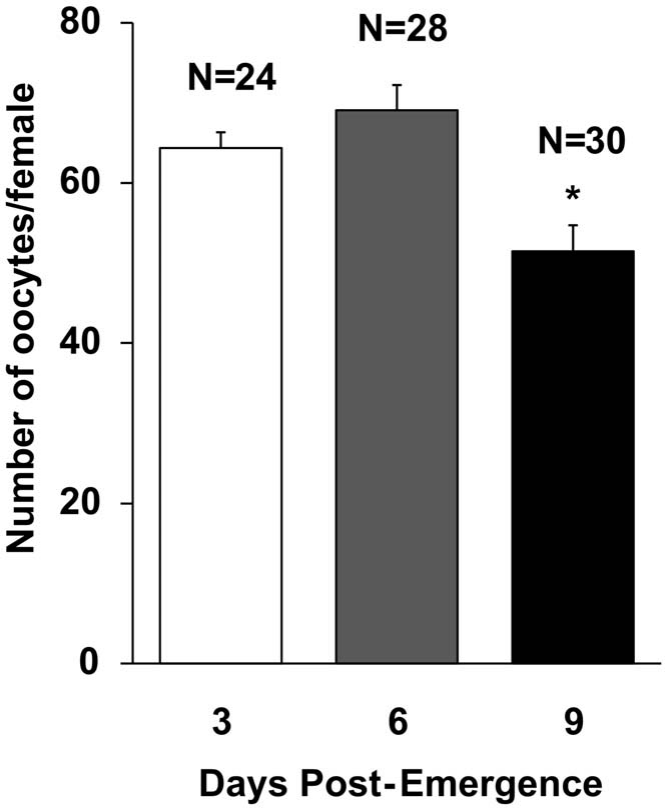
Effect of age at blood feed on subsequent fecundity of female *Lu. longipalpis*. Bars represents average number of oocytes dissected 5 days after blood meal ± SEM. Sand flies were blood-fed at 3, 6 and 9 days Post-Emergence. Asterisk indicates statistical difference at P<0.005 (ANOVA). Results represent two independent biological replicates.

To evaluate the effect of ageing in fecundity, females of different age groups were blood fed and dissected to examine difference in developing oocyte numbers. Female *Lu. longipalpis* from the older age group showed a decrease in the number of developing oocytes dissected five days after blood feeding in comparison to younger sand flies(fig. 1). Female *Lu. longipalpis* that were bloodfed 3 and 6 days post-emergence (PE) showed no significant difference in oocyte numbers. However, sand flies bloodfed at 9 days PE showed a significant decrease in oocyte numbers after dissecting 5 days after blood feeding (fig. 1; P<0.005, ANOVA).

### ROS-scavenging reverses age related loss of fecundity

**Figure 2:**
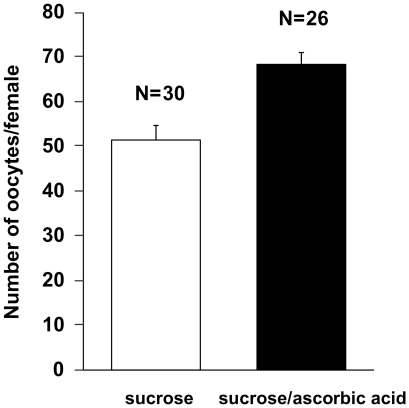
Effect of ascorbic acid supplementation on fecundity in *Lu. longipalpis*. Flies were blood-fed 9 days Post-Emergence and bar chart represents average number of oocytes dissected 5 days after blood meal ± SEM (combined samples derived from 2 independent experiments). Sand flies fed on 20 mM ascorbic acid-supplemented 70% sucrose solution show significantly higher oocyte numbers in comparison to control sand flies (P<0.0001, *t*-test).

To evaluate the role of ROS scavenging in age-related decrease of fecundity, 9 day old female *Lu. longipalpis* were fed a sucrose meal supplemented with a ROS-scavenger upon emergence until end of the experiment. Sand flies were offered a 70% sucrose solution supplemented with 20 mM ascorbic acid and subsequently blood-fed on day 9 PE. 20 mM ascorbic acid was chosen after evaluating mortality of sand flies when offered 100, 50, 20, 10 and 5 mM ascorbic acid in 70% sucrose (data not shown). The number of developing oocytes dissected 5 days after blood feeding was significantly higher (fig. 2; P<0.0001, t-test) in sand flies that received a sugar meal supplemented with 20 mM ascorbic acid in comparison to control sand flies fed on 70% sucrose solution. This suggests that exogenous ROS-scavenging can reverse age-related loss of fecundity in sand flies blood fed 9 days PE.

### Catalase activity is reduced in developing oocytes of older flies and ROS scavengers reverse catalase depletion

**Figure 3:**
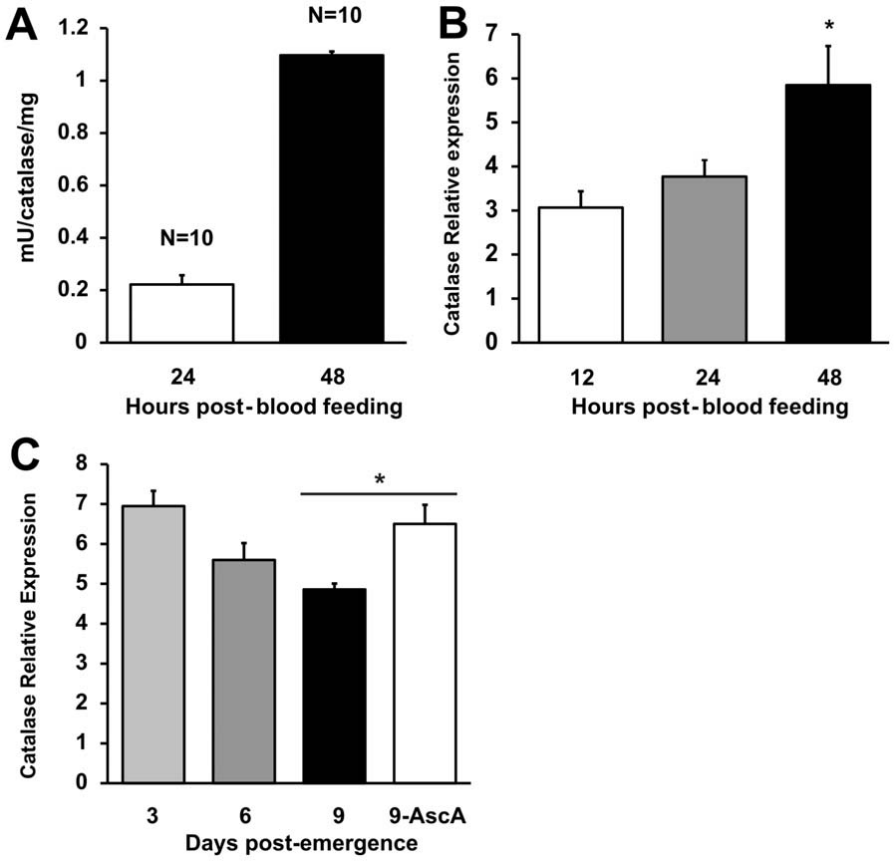
Changes in catalase in the developing oocyte of *Lu. longipalpis*. (A) Catalase activity of developing oocytes after blood feeding. Six day old female *Lu. longipalpis* were blood-fed and dissected at 24 and 48 hours. Enzymatic activity in the developing oocytes was significantly higher at 48 hours compared to 24 hours after blood feeding (*P*<0.0001 , *T*-test). Bar charts represent mean ± SE of combined samples from 2 independent experiments. (B) Relative expression of catalase LlongKat1 mRNA in developing oocytes dissected at 12, 24 and 48 hours from 6 days-old blood-fed female *Lu longipalpis*, (*n* = three groups of 20 females each). Asterisk indicates statistical difference at P<0.05 (ANOVA). Bar charts represent mean ± SEM of combined samples from 2 independent experiments. (C) Age-related decrease of catalase mRNA relative expression in developing oocyte. Flies were blood-fed at 3, 6 and 9 days Post-Emergence (*n* = three groups of 15 females each) and whole ovaries were dissected 48 hours after blood feeding. Relative expression was statistically different in all 3, 6 and 9 days old flies (P = 0.001, ANOVA). A 4^th^ group (n = 15 females) fed on an ascorbic acid-supplemented sugar solution upon emergence (9-AscA) showed catalase relative expression levels similar to groups of younger flies fed on 70% sucrose solution, and statistically higher than the non-treated, 9 DPE group (P<0.002, t-test). Bar charts represent mean ± SEM of combined samples from 2 independent experiments.

Flies were assayed at 24 h and 48 h to find out if catalase accumulated in developing oocytes. Ovaries of *Lu. longipalpis* dissected 6 days PE contained higher catalase enzymatic activity at 48 h compared to 24 h after blood feeding (fig. 3A; P<0.0001 , t-test). Moreover, mRNA expression of catalase increased with oocyte development from 12 to 48 hours after blood feeding (fig. 3B).

To further understand the role of endogenous ROS-scavenging and ageing, flies from different age groups were assayed for catalase LlonKat1 expression. Flies from different age groups (3, 6 and 9 days PE) showed a decrease in expression in ovaries dissected at 48 hours after blood feeding (fig 3c; P = 0.001, ANOVA). Interestingly, when 9 day old sand flies were fed with a 20 mM ascorbic acid supplemented sugar meal, catalase LlonKat1 mRNA expression was significantly higher compared to flies of the same age fed on sucrose only (fig 3c; P<0.002, t-test). The results show that a) catalase accumulates in the developing oocyte as shown by increase in enzymatic activity and relative expression, b) catalase expression is age-dependant and is lower in older flies and c) the dietary supplementation with an exogenous ROS-scavenger increases catalase expression in older flies.

**Figure 4:**
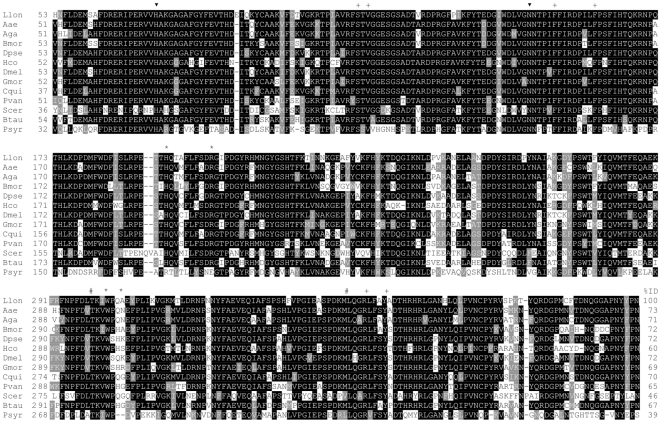
Amino acid sequence alignment of selected catalases. Sequences were retrieved from GenBank (GB), Protein Data Bank (PDB) or from Peroxibase (PB). The listed proteins are respectively from *Lutzomyia longipalpis* (GB:ABV60342.1), *Aedes aegypti* (PB:5267), *Anopheles gambiae* (PB:5269), *Bombyx mori* (PB:5266), *Drosophila pseudoobscura* (PB:5273), *Haemonchus contortus* (PB:5270), *D. melanogaster* (GB:NP_536731.1), *Glossina morsitans morsitans* (GB:ADD20421.1), *Culex quinquefasciatus* (GB:XP_001848573.1), *Penaeus vannamei* (PB:5278), *Saccharomyces cerevisiae* (PDB:1A4E), *Bos taurus* (PDB:8CAT), *Pseudomonas syringae* (PDB:1M7S). Conserved residues in catalases are with black background, consensus alternatives are shaded. The symbols ▾, +, and * mark catalytic, heme binding and NADPH binding residues, respectively. The symbol # mark residues that define heme orientation. All sequences are from clade 3 of monofunctional catalases, with the exception of Psyr, which is a clade 2 enzyme. In catalases from clade 2 (Psyr numbering), heme orientation (His-IV) is defined by residues 301 (never Leu) and 350 (frequently Leu). In catalases from clade 3, these positions are commonly occupied by Leu and non-Leucine residues, respectively. NADPH binding catalases have the signature (Btau numbering) His 193, Arg 202, Val 301 and His 304, which is not present in catalases from clades 1 (not shown) and 2 (Psyr). Insect catalases share some of the NADPH binding residues, but not all. However, catalytic residues and heme binding residues are fully conserved in all sequences.

*Lutzomyia longipalpis* catalase sequence was already described [26], [27] and was retrieved from the GenBank (ABV60342.1). It codes for a protein (named LlonKat1 in this study) with molecular mass of 57682 Da and isoelectric point of 8.28, without a signal peptide and mitochondrial or peroxisomal targeting sequences of types 1 and 2. LlonKat1 has high identity (ranging from 46–73%) to catalase sequences from other insects, crustaceans, yeast and mammals and lower identity to the bacterial catalase from *Pseudomonas syringae* (fig. 4). LlonKat1 sequence contains the conserved residues His73 and Asn147 (catalytic), Ser113, Val115, Phe152, Phe160, Leu298, Met349, Arg353, Tyr357 (heme binding/coordination) and His193, Arg202, Ile301, Gln304 (putative NADPH binding pocket) (fig. 4).

### Catalase gene RNAi mediated depletion leads to a decrease in sand fly fecundity

**Figure 5:**
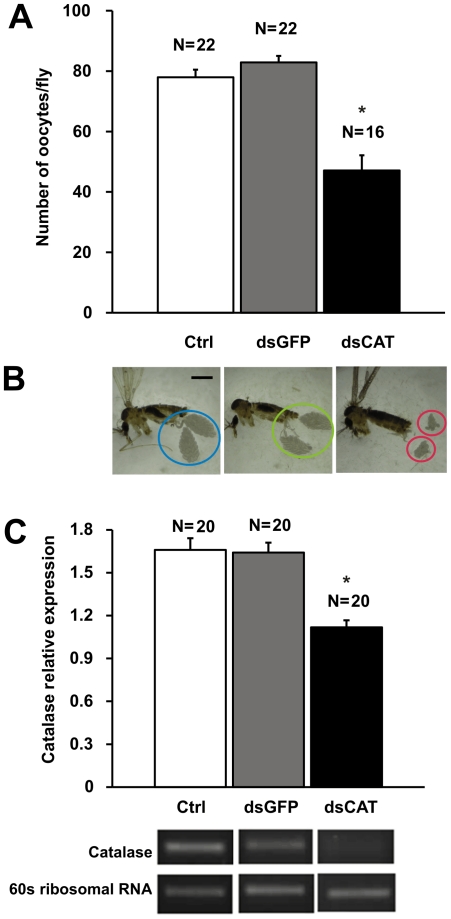
RNAi-mediated depletion of catalase LlonKat1 in female *Lu. Longipalpis* and its effect on fecundity. (A) Average number of developing oocytes dissected 48 hours days after blood meal ± SEM (combined samples derived from 2 independent experiments). Asterisk indicates statistical significance at P<0.005(ANOVA). (B) Relative development of female *Lu. longipalpis* ovaries observed upon catalase gene knockdown by RNAi, in comparison to mock-injected and uninjected control sand flies. Bar = 1 mm. (C) Relative expression of catalase LlongKat1 mRNA in whole fly homogenates from dsRNA-injected catalase knock-down sand flies. Bar charts represent mean ± SEM of combined samples from at least 2 independent experiments. Asterisk indicates statistical difference at P<0.05 (ANOVA).

The gene sequence of *Lu. longipalpis* catalase was obtained from a cDNA library constructed from sand fly whole bodies (NSFM-142e04.q1k [27]) and aligned with a previous described catalase obtained from *Lu. longipalpis* midguts (GenBank Accession number: EU124624.1) , showing a high level of identity (99%) and similarity (99%) (fig. S1). As any other sequence from *Lu. longipalpis* was identified as putative catalase, either in the whole body or midgut cDNA libraries, this gene is probably a single copy gene in *Lu. longipalpis*. To confirm the role of endogenous ROS-scavenging in fecundity catalase was depleted using RNAi. Flies injected with 144 ng of dsRNA for catalase (dsCAT) showed a dramatic decrease in oocyte number dissected 48 hours after blood feeding (fig. 5A; P<0.005, ANOVA) compared to sand flies injected with a non-related dsRNA (dsGFP) and uninjected sand flies. A change in appearance of ovaries was observed during dissections with matured ovaries, in sand flies injected with dsRNA for catalase, appearing underdeveloped in comparison to both mock-injected and uninjected controls (fig. 5B). A dsRNA-mediated significant reduction in catalase expression in whole flies was observed by RT-PCR (fig. 5C), with no effects on the catalase expression in the midgut (data not shown). These results confirm that endogenous ROS-scavenging in developing oocytes plays a major role in female *Lu. longipalpis* fecundity.

### Effect of ROS-scavenging in the survival of sand flies

**Figure 6:**
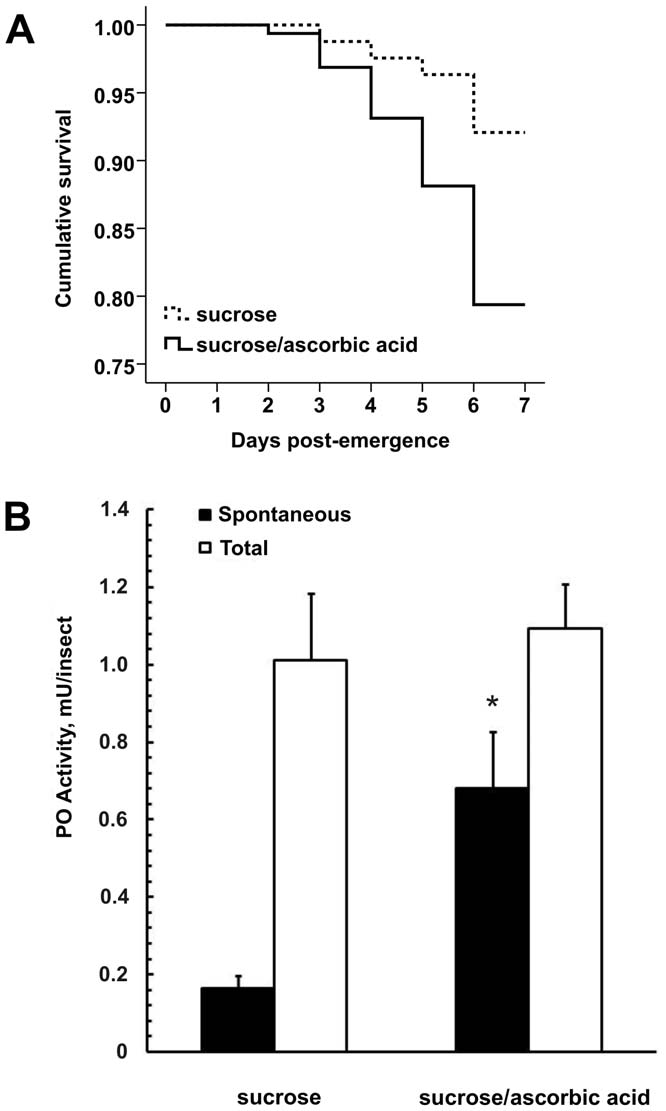
Effect of dietary supplementation of ascorbic acid on mortality of sugar fed *Lu. longipalpis*. (**A**) Female sand flies were offered a 70% sucrose solution supplemented with 20 mM ascorbic acid or a non-supplemented sucrose solution. Experimental flies (sucrose +20 mM ascorbic acid) exhibited a significantly lower survival rate compared to control flies, (*p*<0.001, Kaplan-Meier, Log Rank χ^2^ test). (**B**) Spontaneous and total phenoloxidase (PO) activity in *Lu. longipalpis* females after 7 days of feeding with 70% sucrose solution or 70% sucrose solution supplemented with 20 mM ascorbic acid. Spontaneous PO activity in ascorbic acid supplemented flies was significantly higher than control flies (P<0.05 , *T*-test). Results are mean ± SEM from 2 independent experiments with 10 sand flies per experiment.

To evaluate the role of exogenous ROS scavenging in survival, female *Lu. longipalpis* were fed with an antioxidant-supplemented sugar meal upon emergence. Mortality was recorded from day 1 PE up to day 7 PE. Survival curves depict an increase in mortality due to exogenous ROS-scavenging by an exogenous antioxidant (fig. 6A). In order to assess whether the higher mortality rate was related to an effect on sand fly immune homeostasis , phenoloxidase (PO) activity was measured in control and antioxidant-supplemented females. Spontaneous PO is defined as the activity measured upon reaction with 3,4 dihydroxy-DL-phenylalanine (DOPA), and corresponds to the enzyme that is already activated in physiological conditions and total activity was the activity observed after *in vitro* activation of the enzyme, by preincubating the sample with bovine trypsin. Sand flies fed on ascorbic acid-supplemented sucrose showed a significant increase in spontaneous PO (fig. 6B; P<0.05 , t-test) but no difference in total PO activity.

**Figure 7:**
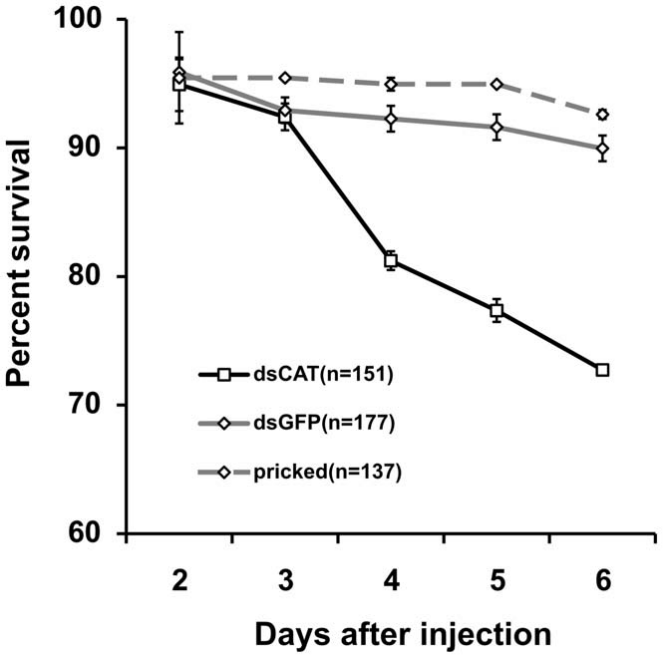
Survival in female *Lu. Longipalpis* after RNAi-mediated depletion of catalase LlonKat1. Experimental group (dsCAT) exhibits a significantly lower survival rate compared to both dsGFP and pricked control groups, (P<0.0001, Kaplan-Meier, Log Rank χ^2^ test). Results represent mean ± SEM of 3 independent biological replicates.

To further investigate if ROS-scavenging was implicated in increased mortality, catalase LlonKat1 was depleted via RNAi injection in female *Lu. longipalpis* and mortality was recorded from day 1 PE up to day 7 PE. Mortality rates was higher in knocked down (dsCAT) sand flies (fig. 7), compared to flies injected with a non-related dsRNA (dsGFP) and non-injected flies. These results show that ROS-scavenging by either endogenous or exogenous antioxidants play an important role in female *Lu. longipalpis* survival.

## Discussion

The present study suggests for the first time that catalase-mediated ROS scavenging has a significant impact on female *Lu. longipalpis* fecundity and survival. Female *Lu. longipalpis* from different age groups showed differences in developing oocytes numbers, with the oldest (9 days PE) presenting the lowest number of oocytes (fig. 1). The age-related loss of fecundity could be reversed with dietary supplementation of a potent exogenous ROS-scavenger (fig. 2). This underlines the importance of catalase in the reproductive success of blood sucking dipterans. Evidence from other dipterans show that aging results in increase of oxidative stress and loss of enzymatic antioxidant efficiency [13], [14], [28]. Moreover, inactivation or silencing of catalase in *Drosophila melanogaster* [29], *Musca domesti*ca [30], *Rhodnius prolixus* [31]and *Anopheles gambiae* [9] led to increased mortality due to increase in ROS levels. It is likely that accumulation of ROS in older flies could account for the decrease of female sand fly fecundity due to an increase in oxidative stress, loss of antioxidant enzymatic efficiency or both. In *An gambiae*, fecundity of female mosquitoes declined with age, with reduction of number of eggs oviposited and number of larvae hatched per female [7]. We did not measure differences in fecundity in terms of larval development but it is likely that the age-related differences in fertility would have resulted in less viable larvae being produced from older flies, as they would be presumably exposed for a longer periods to oxidative damage.

Catalase enzymatic activity as well as catalase LlonKat1 mRNA relative expression increased in the ovaries of older female sand flies (6 days PE) after the blood feeding (figs. 3a and b). Protein expression and accumulation increased upon blood feeding in maturing ovaries of mosquitoes due to nutrient allocation for egg production [32], [33]. It has been shown in different insect species that antioxidant activity increases in the ovaries to protect the embryo from oxidative damage [34], [35]. It is conceivable that such accumulation of catalase in sand fly ovaries also provides the means to protect developing eggs from oxidative damage. Additional support for this hypothesis was given by the dramatic decrease in developing oocyte numbers upon successful catalase gene depletion by RNAi in female sand flies (fig. 5A and b).

Interestingly, oral delivery of ascorbic acid seemed to stimulate catalase LlonKat1 mRNA expression in older flies to levels similar to that of younger flies (fig. 3C). It has been shown that age-related accumulation of ROS/oxidative stress leads to loss of efficiency in cellular processes [13], [14], [15], [28], [36], therefore it is possible that ROS-scavenging by an exogenous antioxidant slowed or lowered such deleterious effects in either catalase LlonKcat1 mRNA or in other molecules involved in its upregulation. On the other hand, it has been shown that ascorbate is a potent inhibitor of catalase [37], the inhibition being independent of substrate concentration and pH and strongly influenced by temperature. Furthermore, catalase incubation with ascorbate leads to degradative changes to the catalase molecule [38]. In our experiments, the increase in catalase gene expression might reflect a compensation response to replenish normal catalase levels in the sand fly body after catalase was degraded, by an unknown mechanism, during ascorbic acid supplementation with the sugar meal.

Catalase LlonKcat1 does not have a signal peptide or targeting sequences to mitochondria or peroxisomes. These features suggest a cytosolic location but this needs confirmation. Based on the identities with other catalases retrieved from Peroxibase [39], LlonKat1 seems to belong to the monofunctional clade 3 of catalases, which includes sequences from bacteria, archaebacteria, protists, fungi, plants and animals. These enzymes have small subunits with molecular mass ranging from 43–75 kDa [40], which is consistent with LlonKat1 monomer predicted molecular mass (57.7 kDa). All conserved catalytic and heme binding residues are present in LlonKat1 sequence, suggesting a full catalytic activity, and the presence of residues Leu298, Met349 indicate that His70 is above the ring III of the heme molecule (His-III orientation), as seen in other clade 3 catalases [41].

ROS-scavenging by dietary supplementation of ascorbic acid (fig. 6a) led to a reduction in sand fly survival. When antioxidants were provided to a susceptible strain of *Anopheles gambiae* to *Plasmodium* infection, a similar but more drastic effect was observed with female mosquitoes [7]. Magwere *et al.* [42] observed that antioxidant supplementation did not extend the lifespan of wild type *Drosophila*. Similarly, Bayne *et al.* [43] showed that overexpression of MnSOD and catalase, despite protecting *Drosophila* from oxidative stress, were detrimental for lifespan and physical fitness of the insects. Kang *et al.* [44] observed a reduction in the lifespan of *Anopheles stephensi* when the mosquitoes were bloodfed with the antioxidant MnTBAP in comparison with the buffer control. It has been hypothesized that a minimal level of ROS might be required to maintain the balance of the gut microbiota and that a baseline level of ROS activity might be crucial for basic midgut physiology. Previous studies done with other dipteran species had showed that ROS release constitutes a first line of defence against pathogens in the midgut [45]. Experiments in *D. melanogaster* have demonstrated the existence of a midgut-specific active ROS releasing system against orally delivered bacteria [8]. In the present study, higher activities of spontaneous PO were recorded and this might be due to an increase in microbial infection associated with sand flies that fed on an ascorbic acid-supplemented sugar meal. In insects, PPO activation is often related to bacterial or fungal infections [45]. Since only the soluble form of PO was measured (see Material and Methods), it is more likely that the activity was related to the immune response rather than to the melanisation of the adult cuticle or egg shell. PO has already been described in gut tissues or adhered hemocytes in other dipterans [46]. It is possible that mortality in our experimental group fed with sucrose supplemented with ascorbic acid may be due to a decrease in ROS production inside the midgut and that ROS activity, similar to the events in certain strains of mosquitoes, may play a role in sand fly immunity towards opportunistic microbes or be involved in important cellular signalling pathways [47], [48].

There is evidence of other antioxidant enzymes with catalase-like functions found in the sand fly midgut, such as peroxiredoxins [26], [27]. These are a family of thioredoxin-dependent peroxidases, found in several insect species [49], [50], [51], [52], that function as ROS-scavengers as well as other cellular processes. However their efficiency in converting H_2_O_2_ was found to be significantly lower compared to catalase [53]. We are currently investigating the role of these antioxidant enzymes in ROS detoxification and fecundity and survival of female sand flies, as well as studies to confirm if proliferation of bacteria due to ROS reduction could account for the differences in sand fly survival.

Recent studies on transgenic *Anopheles stephensi* (the leading malaria vector in India and parts of Asia and the Middle East) overexpressing the protein kinase AKT gene increased the insulin signalling in the mosquito midgut, significantly reducing mosquito lifespan and inhibiting *P. falciparum* development [54]. The role of genes involved in stress responses in *Plasmodium* survival within the mosquito midgut was investigated by Jaramillo-Gutierrez et al. [55]. RNAi gene knockdown of the OXR1 gene (oxidation resistance gene) in *Anopheles gambiae* showed that this gene regulates the basal levels of catalase and glutathione peroxidase expression and that OXR1 gene knockdown decreased *Plasmodium berghei* oocyst formation. The finding of a *Lu. longipalpis* OXR1 gene homologue (unpublished) will shed some light into the regulation of ROS production within the sand fly gut and will also help to understand how ROS production impacts *Leishmania* development in the sand fly midgut.

Current vector control strategies rely on spraying of residual insecticides to control vector population. Insect transgenesis and paratransgenesis are novel strategies that aim at reducing insect vectorial capacity by using genetic manipulation of disease vectors, rendering them incapable or less efficient to transmit a given pathogen [56] or even reducing the longevity and fecundity of a given insect vector. This study shows that catalase is a key gene in determining survival and fecundity of phlebotomine sand flies and future developments may warrant this gene being included as a potential target to reduce female sand fly fitness and reproductive capacity in the field.

## Materials and Methods

### Insects

All experiments were carried out using insectary-reared *Lu. longipalpis* from a colony first started with individuals caught in Jacobina, Brazil. Insects were kept under standard laboratory conditions [57]. Insects were fed with 70% w/v sucrose solution in cotton wool (unless stated differently in experiments), kept under a photoperiod of 8 hours light/16 hours darkness, temperature of 27°C (±2) and a relative humidity of >80% inside the rearing cages. Rabbit blood feeding was via a Hemotek membrane feeder (Discovery Workshops, UK) at 37°C. All procedures involving animals were performed in accordance with UK Government (Home Office) and EC regulations.

### Fecundity assays

Female *Lu. longipalpis* were allowed to mate under regular rearing conditions and fed with rabbit blood at three, six and nine days post-emergence (DPE). A batch of >500 flies was released into a large (20 m^3^) rearing cage and groups of ∼100 individuals were transferred to medium sized cages (5 m^3^) at 3, 6 and 9 DPE and blood-fed as above. Fifteen fully-engorged females were then transferred to a new medium rearing cage. Insects were dissected five days later to count developing oocytes.

### ROS-scavenger feeding

Fecundity assays were carried out as described above with female *Lu. longipalpis* fed on a 70% sucrose solution supplemented with 20 mM ascorbic acid and blood-fed at 9 DPE. Supplemented sucrose-meal was freshly changed daily and continued after blood-feeding. A 9 DPE control group was reared under the same conditions but fed with a 70% sucrose solution. Only fully engorged insects from both groups were selected for the experiments.

### Ovarian Catalase Activity

Ovaries were collected from 5 female sand flies at 24 and 48 hrs post blood feeding (PBF). Samples were homogenised in 50 ul of 0.15 M NaCl solution, kept on ice and transferred to a −80°C freezer until needed. Before assays, samples were centrifuged at 5000 RPM for 2 minutes and 1 µl of the supernatant was diluted in 24.9 µl of 0.15 M NaCl solution. Catalase activity was determined using Amplex Red Catalase Assay Kit (Invitrogen Ltd) following the manufacture's protocol. Enzyme-specific activities were expressed as units/mg of protein. One unit of catalase activity was defined as 1 µM of H_2_O_2_ consumed per minute. All assays were carried out in triplicate. Fluorescence was measured using a Varioskan fluorescence spectrometer (Thermo Electron) with an excitation wavelength of 560 nm and an emission wavelength of 590 nm. Ovarian catalase activity was normalised using the total amount of protein in the whole body (minus dissected ovaries) using the BIORAD® Protein assay reagent following the manufacturer's protocol and using bovine serum protein as standard. Endpoint absorbance was measured at 595 nm in a 96 well plate with a microplate reader (VersaMax Microplate Reader, Molecular Devices Inc.).

### Ovarian Catalase Expression

Six DPE sand flies were blood fed and ovaries from 10 sand flies (two pools of 5 flies) were dissected at 12, 24 and 48 hours PBF, homogenised in 50 µl of TRI Reagent® (Ambion, Austin, TX) and kept at −80°C until needed. RNA was extracted following the manufacturer's protocol. Total RNA was quantified using a Nanodrop®(NanoDrop Technologies, Wilmington, USA) and normalised to 10 ng/µl. RT-PCR was carried out with SuperScript® III One-Step RT-PCR System with Platinum® Taq DNA Polymerase Kit (Invitrogen, San Diego, CA) performing 19 cycles and following the manufacture's protocol (primers listed on table 1). Relative expression of catalase was normalised using a housekeeping gene (AM088777, 60S ribosomal protein L3). RT-PCR products were analysed by 1.5% agarose/ethidium bromide gel electrophoresis and reduction in catalase expression was determined by densitometric measurement of bands using the softwares GeneSnap/GeneTools (Syngene, UK).

**Table 1:** Oligonucleotides for dsRNA synthesis and Reverse Transcriptase PCR.

Oligonucleotide	5′-3′sequence	Size (bp)
**dsCAT484 Forward**	**TAATACGACTCACTATAGGG**TGTTGCAGGGACGTCTCTTTGCC	524
**dsCAT484 reverse**	**TAATACGACTCACTATAGGG**AGGTTGGAGCACTTCTTGCGTTCG	
**dsGFP Forward**	**TAATACGACTCACTATAGGG**ACGTAAACGGCCACAAGTTC	693
**dsGFP Reverse**	**TAATACGACTCACTATAGGG**CTTGTACAGCTCGTCCATGCC	
**RT CAT484 Forward**	TGTTGCAGGGACGTCTCTTTGCC	484
**RT CAT484 Reverse**	AGGTTGGAGCACTTCTTGCGTTCG	
**RT Ribo60S Forward**	TCTCATCGGAAGTTTTCTGC	850
**RT Ribo60 Reverse**	GGCTTGTGACACCCTTGAAT	

### Age-related expression of ovarian catalase

To measure catalase LlongKat1 mRNA expression levels in different age groups, 3, 6 and 9 DPE sand flies were blood fed and ovaries from 10 sand flies (two pools of 5 flies) were dissected at 48 hours PBF. Additionally, to evaluate the effect of feeding a ROS-scavenger in age-related expression of ovarian catalase, a group of 9 days old sand flies was fed with ascorbic acid-supplemented sucrose solution as described above, blood fed and dissected at 48 hours. RNA was extracted and checked for catalase relative expression as above.

### RNAi-mediated catalase knockdown

Sense and anti-sense catalase-specific primers flanked by the T7 promoter site (Table 1) PCR amplified a 484 bp product from a plasmid obtained from a whole body Lu. longipalpis normalised cDNA library [27] that was used as template for double-stranded RNA synthesis dsRNA. Transcription reactions and column purification were carried out using the Megascript RNAi Kit (Ambion®) following the manufacturer's protocol. dsRNA purity was assessed by 1.5% agarose/ethidium bromide gel electrophoresis and dsRNA was quantitated using a Nanodrop ND-1000 Spectrophotometer (LabTech, UK). dsRNA was eluted with nuclease-free water at 65°C, concentrated to 4.5 µg/µL with a Christ® RVC 2–25 rotational vacuum concentrator and stored at −80°C until needed. Enhanced Green Fluorescent protein (eGFP) dsRNA was produced from a 653 bp amplicon of the pEGFP-N1 expression plasmid (Clontech) and used as a ‘mock’ injected control. RNAi was achieved by dsRNA injections as previously described [58]. After injections, sand flies were transferred to cages and kept with access to 70% sucrose solution *ad libitum*. Developing oocytes were dissected and counted 48 hours after blood feeding. Non-injected flies of the same age and kept under the same conditions were used as second control. Three pools of three whole sand flies were collected from each group to evaluate knockdown by RT-PCR.

### Survival assays

To assess sand fly survival mediated by ROS-scavenging related to catalase activity, RNAi-mediated catalase knock down was carried out in a group of 50 sand flies. Flies were injected with dsRNA for catalase (dsCAT) as described above. To exclude wound-related mortality, all dead flies at 24 hrs post-injections were removed and were not included in the experiment. Dead sand flies were counted and removed from the cage daily from day 2 to 7 after injection. Flies injected with dsRNA for GFP (dsGFP) and a needle-pricked group were used as controls. To assess exogenous ROS-scavenging related survival, 50 female *Lu. longipalpis* were collected upon emergence and sugar fed on a 70% w/v sucrose solution supplemented with 20 mM ascorbic acid. Dead sand flies were counted and removed from the cage every day until day seven. A group of sand flies fed with 70% sucrose was used as a control.

### Phenoloxidase assays

Phenoloxidase activity was determined by measuring the production of dopachrome from 3,4 dihydroxy-DL-phenylalanine (DOPA) [59], [60]. Briefly, single flies were homogenized in 60 µL of PBS and centrifuged at 25,000 g for 5 min at 4°C to recover the soluble fraction. 20 µL of supernatant was mixed with 10 µL of PBS (spontaneous PO) or trypsin solution (for total PO activity; 1 mg/mL in PBS, FLUKA cat. no. 93614), incubated for 20 min at 37°C followed by the addition of 20 µL of a saturated solution of DOPA (4 mg/mL in PBS) and absorbance (490 nm) measured by kinetic assay for 1 h at 5 minutes intervals in a microplate reader at 30°C.

The PO activity was measured to ensure that activity was proportional to protein concentration and incubation time. Independent experiments showed that the PO activity was stable in the conditions above. Controls with no enzyme or no substrate were included. One unit of enzyme (U) is defined as the amount that produces 0.001 unit of absorbance/min.

### Sequence analysis

The coding sequence of LlonKat1 was analyzed using the algorithms pI/Mw tool [61] , signal IP [62], PTS1 Predictor [63], PeroxiP [64], TargetP [62] based at the EXPASY Proteomics Server (http://expasy.org/). Selected amino acid sequences of catalases were aligned with catalase LlonKat1 using the ClustalW Multiple Alignment tool in BioEdit Sequence Alignment Editor (http://www.mbio.ncsu.edu/BioEdit/BioEdit.html). Alignment was generated using Boxshade (http://www.ch.embnet.org/software/BOX_form.html).

### Statistical analysis

Comparisons between means of two independent groups were carried put using a pair-wise t-test. Multiple comparisons were done by one-way ANOVA. Survival curves were analyzed with the Kaplan-Meier Log Rank χ^2^ test. Significance was considered when P<0.05. All data were analysed with the use of the SPSS Data Editor software (version 17.0, SPSS Inc).

## Supporting Information

Figure S1 **Structure-based alignment of the aminoacid sequence of** ***Lutzomyia longipalpis*** **catalase, translated from a whole body (GeneDB NSFM-142e04.q1k** [**27**] **) and a midgut-specific (GenBank Accession number: EU124624.1** [**26**] **) cDNA library.** Sequences show a 99% identity and a 99% similarity. > Represents the targeted region for dsRNA-mediated gene silencing.
(DOC)
