# Supplementary material for: Reactive Oxygen Species Scavenging by Catalase Is Important for Female Lutzomyia longipalpis Fecundity and Mortality
Source: PLoS One. 2011 Mar 9;6(3):e17486. doi: 10.1371/journal.pone.0017486 (PMC3052318; doi:10.1371/journal.pone.0017486)
Supplement: Figure S1 — Structure-based alignment of the aminoacid sequence of Lutzomyia longipalpis catalase, translated from a whole body (GeneDB NSFM-142e04.q1k [27] ) and a midgut-specific (GenBank Accession number: EU124624.1 [26] ) cDNA library. Sequences show a 99% identity and a 99% similarity. > Represents the targeted region for dsRNA-mediated gene silencing. (DOC) [file pone.0017486.s001.doc]

whole_body 1 MSARGPAEDQLKLYKKSQKGATSTVTTKHGAPIGVRSAAQTVGLNGPILLQDHNFLDEMS
midgut 1 MSARGPAEDQLKLYKKSQKGATSTVTTKHGAPIGVRSAAQTVGLNGPILLQDHNFLDEMS


whole_body 61 AFDRERIPERVVHAKGAGAFGYFEVTHDEIQKYCAAKVFDTVGKRTPIAVRFSTVGGESG
midgut 61 AFDRERIPERVVHAKGAGAFGYFEVTHDEIQKYCAAKVFDTVGKRTPIAVRFSTVGGESG


whole_body 121 SADTVRDPRGFPIKFYTEDGIWDLVGNNTPIFFIRDPILFPSFIHTQKRNPQTHLKDPDM
midgut 121 SADTVRDPRGFPIKFYTEDGIWDLVGNNTPIFFIRDPILFPSFIHTQKRNPQTHLKDPDM


whole_body 181 FWDFISLRPETTHQTAFLFSDRGIPDGYRHMNGYGSHTFKTINNKGEAFYVKFHYKTDQG
midgut 181 FWDFISLRPETTHQTAFLFSDRGIPDGYRHMNGYGSHTFKTINNKGEAFYVKFHYKTDQG


whole_body 241 IKNLDPVKANELAANDPDYSIRDLYNAIAKGDYPSWTFYIQVMTFEQAEKFRFNPFDLTK
midgut 241 IKNLDPVKANELAANDPDYSIRDPYNAIAKGDYPSWTFYIQVMTFEQAEKFRFNPFDLTK

 >>>>>>>>>>>>>>>>
whole_body 301 IWPQAEYPLIKVGKMTLDRNPNNYFAEVEQIAFSPSHFVPGIEASPDKMLQGRLFAYADT
midgut 301 IWPQAEYPLIKVGKMTLDRNPNNYFAEVEQIAFSPSHFVPGIEASPDKMLQGRLFAYADT

 >>>>>>>>>>>>>>>>>>>>>>>>>>>>>>>>>>>>>>>>>>>>>>>>>>>>>>>>>>>>
whole_body 361 HRHRLGANHLQLPVNCPYRVSPKTYQRDGPMCFTDNQGGAPNYYPNSFAGPDTCPRALKL
midgut 361 HRHRLGANHLQLPVNCPYRVSPKTYQRDGPMCFTDNQGGAPNYYPNSFAGPDTCPRALKL

 >>>>>>>>>>>>>>>>>>>>>>>>>>>>>>>>>>>>>>>>>>>>>>>>>>>>>>>>>>>>
whole_body 421 NPPYKICGDVARFDSGETEDNYAQVTDFYRRVLDAPARERLAQNIAGHLCAASQFIQERA
midgut 421 NPPYKICGDVARFDSGETEDNYAQVTDFYRRVLDAPARERLAQNIAGHLCAASQFIQERA

 >>>>>>>>>>>>>>>>>>>>>>>>>>>>>>
whole_body 481 VKNFSQVDASLGQKLTELLNMYERKKCSNL
midgut 481 VKNFSQVDASLGQKLTELLNMYERKKCSNL
